# Promotion of Medical Science

**Published:** 1854-05

**Authors:** 


### ART. VI.—*Promotion of Medical Science.*

The speech of Mr. Germain of this city, which we give below, is worthy of a place in medical literature. It is by such arguments, advanced by such men as Mr. Germain, and others equally able and patriotic, that we have at last secured to us the privilege of legally pursuing the study of anatomy.

The present bill, though very imperfect, is still valuable, if we look at it only as an entering wedge to more liberal legislation. We believe that the friends of the bill in the Legislature did all they were able, to make it acceptable to the profession, but very much had to be conceded to the ignorant and fanatic portion of that body, in order to secure the passage of any bill. H.

*Speech of Mr. Germain, of Erie, delivered in the Assembly, in Committee of the Whole, February 28th, upon the Bill "For the Promotion of Medical Science."*

Mr. Germain—I believe it is not strictly in order to discuss the general merits of a bill in committee of the whole until after it has been gone through with by sections. But as the first section of this bill, now engaging the attention of the committee, involves the whole merits of the bill, and as those who have preceded me in this debate, have addressed themselves to its general merits, I hope I shall not be deemed out of order, if I shall follow their example. I have apprehended from the tenor of the remarks of those who have spoken in opposition to the bill, that they did not clearly understand its provisions. And that we may not labor under any misapprehension, in relation thereto, I beg leave to call the attention of the committee, particularly, to the provisions of the first section. I will not detain the committee by reading it, but simply state that it provides that no bodies of the dead shall be delivered up to our medical schools for dissection, that shall be claimed by any friends of the deceased within twenty-four hours after his death; or of any that shall be known to have friends even, nor of any who may have expressed a contrary wish during their last sickness. And by an amendment proposed by the gentleman from New York, (Mr. Conkling,) which I hope will be engrafted upon this section, the bodies of all those that shall be delivered up to the medical schools, shall, after the process of dissection shall have been accomplished upon them, be placed in coffins, prepared for the purpose, and decently interred. The gentleman from Tompkins, (Mr. Joy,) who has just taken his seat, in closing his remarks, with all that fervid eloquence which characterizes whatever he says upon this floor, has expressed to us his most earnest wish that this bill may not become a law. With equal sincerity I hope that this bill *may* become a law; that is, if it is fitted to accomplish the purpose for which it is designed. Our laws, relating to surgery, are not creditable to the wisdom of past legislation. The successful practice of surgery is a high art — there is none more so — requiring a minute and thorough knowledge of the organism of the human body, in all its various changes from infancy to old age — in sickness and in health.

The surgeon must see, with his professional eye, the position, size and form of every bone, muscle, vein and artery — the complex web of the nervous system, and the morbid and healthful relation of all these to each other. There should be traced upon his memory, in characters ever legible, a perfect picture of substantive man; so limning his outward and inward being that all its curious and intricate forms, relations and dependencies shall appear. For the instances are not rare, where the surgeon's duties will not give him time to acquire, if he does not possess it, the requisite anatomical information for the case in hand. Some sudden calamity has cast a life, or perchance a score of them, upon the verge of death, to be rescued only by the surgeon's prompt and skillful hand — a hand that can guide the knife through living flesh, mid vital chords and trembling sinews, with unerring certainty. The mechanic who should attempt to repair a complicated machine, without knowing the parts of which it is composed — their various uses and relations to each other — would most likely render it worse rather than better. We would not employ a shoemaker to mend a watch, nor a tailor to repair a steam engine. But how simple are these, and how simple is the most complicated specimen of human art, compared with the mechanism of the human body? We are indeed "fearfully and wonderfully made." To attain unto a full knowledge of the mechanism of the human frame, even under the most favorable circumstances for investigation and study, will tax to the utmost, as it is conceded, the powers of any one mind, however gifted. It is only by dividing this great study into many subdivisions, that it can be readily mastered. One may devote years to the investigation and treasuring up in the memory, all the curious forms and relations of the muscular system; another to the nervous; another to the arterial; another to the form and structure of the bones in all their various stages of development; another to the wonders of the respiratory, digestive and secretive organs, not only in their healthful, but in their greatly diversified morbid actions and relations.

Such knowledge, it is conceded by all conversant with the subject, cannot be acquired, even to a respectable extent, from the study of books, plates, or from the most perfect models, the productions of human art; nor in any other way, than by the study of the human body, through the aid of dissection. Inimitable, like all of the handy-works of the great Creator, it can only be thoroughly known and comprehended by a minute and laborious investigation of the thing itself. But upon such knowledge the health, the physical power and activity, and the life even of thousands in our midst are continually dependent. We all see and acknowledge this. Our laws, appreciating this, and sternly requiring its possession by the surgeon, punish him with high penalties for mal-practice, if any suffer through his want of it. But at the same time they deny to him, except to a very limited extent, under appalling punishment, the only possible method by which he can obtain the requisite information.

A Roman tyrant, it is said, while he enforced his laws with merciless severity, hung his edicts at the top of high pillars, where his unhappy people could not read them. Between the spirit of our laws in this respect, and the temper of the Roman butcher, we must acknowledge, with humiliation, there is too close a parallel.

This should not be. If it is inhuman, immoral, or unjust, to resort to the only method by which this knowledge can be obtained, then our laws are wicked and tyrannical that require it. It must be wrong to require what it is wrong to acquire.

Let us repeal all laws punishing the surgeon for mal-practice, or else open to him the only door to that field of knowledge, where alone he can gather up the information that shall enable him to become skillful in his high art. Such a repeal would take from our laws odious features of injustice. But by such a repeal we should be legislating backwards.

Such legislation would be based upon the supposition, that the knowledge of which we speak is either useless, or that the community require no protection against surgical quackery. Both of which are manifestly absurd. The evil is in imposing formidable barriers to the attainment of a most useful science; not in punishing the quack, but in hindering him from becoming an adept in his art. But how can the medical faculty be furnished with a sufficient number of bodies for dissection? This subject is not without its difficulties. The sanctity that surrounds the grave is not a vulgar prejudice. None but a brute could treat with contumely the ashes of the dead. Not that the dead are injured. This is not pretended. The injury is to the human brute, who is thus more imbrutalized.

But in dissection for the acquisition of anatomical science, there is not necessarily, or naturally, any thing of this. The student is intent upon perfecting himself for the practice of a humane and honorable profession. And the loathsome duty of the dissecting room can minister to no unhallowed feeling. And in minutely investigating the complex structure of the most cunning of the Creator's works, feelings of respect and admiration must be for more natural than those of contempt or outrage.

But this bill will not be resisted upon the plea that the dead will be injured; nor upon the other ground, that it will imbrutalize the student of surgery himself to freely practice dissection; and, therefore, that the preservation of the of the public morals requires that he should be withheld therefrom.

If, then, there is no injury that can be done to the dead, nor to the student of surgery, but on the contrary, great benefit to the latter, and great good to all who may require surgical aid, it follows that this bill should become a law, without injury shall be done elsewhere.

Were the feelings of friends to be outraged, or a high moral tone in the people to be lowered or debased thereby, great as the necessity is for such surgical knowledge as this bill would promote, I should feel constrained to oppose it. But the sensibilities of friends are fully protected, as are also the feelings of the sick. For the bodies of those shall not be delivered to the medical schools that shall be claimed by friends; nor of those who knowingly had friends at the time of their decease; nor of those who have expressed a contrary wish.

But an influence is brought to bear against this bill, claiming to be of a religious character. Circular after circular has been addressed to each one of us, upon this subject, solomnly reminding us of man's high destiny in that world which is to come; that "this mortal shall put on immortality," or, in the sublime language of Job, "though worms destroy this body, yet in my flesh shall I see God." These are great words. They raise the man above himself. When I read or think of them, "I feel myself exalted." Far from disregarding these sublime truths, I would inculcate them with all my feeble powers. But I cannot see how this question can in the slightest manner be affected thereby. It cannot be contended that the knife of the surgeon, any more than the maw of the worm, can change man's high destiny. Were it not for giving a ludicrous turn to so serious and important a subject, it might be asked with great pertinency, if it would have been any the less true, if Job had said, "though doctors destroy this body," &c.

Such objections evince too limited views of the great economy of nature, and the power and wisdom of Him at whose "presence the earth shook and trembled;" and they border too nearly upon a groveling materialism to be worthy of much consideration. Every reflecting mind must see that the Creator has intended no such guards as are here indicated to be thrown around man's material mould. He requires not the feeble efforts of man to protect our dust for the great resurrection. But he has surrounded us with many physical evils, warring our destruction. Against many of them, "remedies are not past finding out," nor beyond our reach. The "bane and antidote are both before" us. The surgeon's art is an advancing science. Many physical evils yield to the skill of the surgeon. How many cases are now easily managed, which a hundred, or even fifty years ago were deemed almost incurable? The physicians upon this floor may answer that. Many there are, we all know. Is the surgeon's high knowledge, then, a forbidden lore? "Get wisdom — understanding," is the command. And the Creator's works are all before us for study and investigation. Not to be opened, I grant you, by a sacilegious hand; nor closed against suffering humanity, struggling to overcome the innumerable evils that beset it.

Some adequate provision for the furnishing of subjects for dissection, is in justice due to the members of a high and honorable profession. We will not employ, nor even respect them, without they are skilled in their high calling. Nor will we suffer them to become such, without making them felons to our laws. This is a refinement in injustice degrading to our civilization. The spirited and ambitious student, to whom is given the choice of being a felon or a dunce — scorning what he believes to be tyranical and stupid legislation — hesitates but little as to which he will choose. The consequence is, that no receptacle for the dead is sacred from his intrusion; nor will it be, though we should crowd penalty upon penalty, until we provide that, by legalized means, these, his great necessities, may be supplied. The members of the medical profession are, in other respects, a law-abiding people. There are none more so. They would rejoice to be suffered to do so in this. I say it with all confidence, their violation of law is enforced by unjust and irreconcilable legislation. We require of them the possession of certain knowledge and skill; and we punish them for their deficiency therein. If they resort to the only means of acquiring this, we threaten them with the felon's stripes and the felon's cell. They have wisely or unwisely preferred to risk the dangers with which we have environed their way to professional knowledge, rather than incur the life-long degradation and chastisement that spring from ignorance and imbecility. And it must be confessed that the community have greatly gained by the skill and knowledge thus surreptitiously acquired. And humanity may rejoice that they have not kept our laws.

The surgical departments of our medical colleges require annually, it is aid, some eighty subjects each. And these they get; at great trouble, risk and expense it may be; but where, few but the practical resurrectionists know. It is pretty well understood, however, that no cemetery is secure from their depredations. This bill will protect the sanctity of the grave, by removing all temptations to its violation. But it is said, and here lies the stress of the argument on the other side, that this protection will be at the expense of the most poor, the most defenseless, and the most unfortunate classes among us. This argument would have force if bodily dissection was indeed an evil to the dead; but not otherwise.

But it may be said in connection with this subject, that the poor and laboring class among us will be by far the most benefitted by this bill. For they, as compared with all others, suffer most from quackery. The sleek, subtle and seductive empiric draws his chief nourishment from the life blood of the poor. They are least able to scan his merits, and detect his hollow pretensions. The rich man, when sore misery falls upon him — requiring high medical or surgical skill to alleviate or remove — can most generally summon to his aid, wherever he may be, the first masters of the profession. But when like distress visits the humble dwelling of the poor, its lowly tenant can exercise but little discrimination or choice. He must take up with the first physician at hand, or that he can get. If he be learned and skilled, it is well. But if he be an empiric, his misfortunes are doubled, and his state is pitiable indeed. For to a life struggle with disease, is superadded the more appalling danger, of a death struggle with a quack.

And who are most apt to be the physicians of the poor? The rich man's son can resort to the world-renowned medical schools of Paris or London, or of other cities of Europe, where there is no lack of means to perfect him in his profession. But the son of the poor man must find his professional knowledge here. And there is great danger that it will be measured by the facilities and advantages for study that our laws suffer him to enjoy.

Those who have possessed and improved superior advantages for professional education, seek mostly the wealthy districts of our cities and towns, where their high attainments command appropriate rewards. But their more humble brethren find their patrons more especially amongst the laboring poor of the cities and rural districts. This bill, whil it will be advantageous to all, is preeminently so for the poor man's doctor, and consequently for the poor themselves.

The gentleman from Jefferson has told us, in opposition to this bill, that he has had occasion to resort to a surgeon in affliction — but that instead of being cured by him, he was made a cripple for life. And this, strange as it may seem, he gives as as reason why we should vote agaist this bill. I

would ask the gentleman, whether his misfortunes are more properly due to a superior professional knowledge and skill in his doctor, or to his great deficiency therein?

The supply of bodies which this bill may furnish will be mainly from those who, while living, were brought to wretchedness by improvidence or crime. Having either afflicted the community by their misdeeds, and burdened the State by their punishment; or having been supported by public alms — by offering up their bodies, to the advancement of a humane science, they will make some returns to those whom they have burdened by their wants, or injured by their crimes.

Though I would not argue this question with any partizan zeal, but rather with the impartiality of a judge, yet I confess to some anxiety that this bill shall become a law. Not only because I consider it eminently just, and highly useful in its effects, but also as honorable to the intelligence and wisdom of this Legislature. It is a step in the advance of civilization and humanization. And every error we shall subdue, every prejudice we may overcome, lifts us somewhat from the darkness and degradation into which our race has been cast, and strengthens us and cheers us onward — struggling to a higher and better life.

